# Is the Way to Fight Cancer Paved with Gold? Metal-Based Carbene Complexes with Multiple and Fascinating Biological Features

**DOI:** 10.3390/ph13050091

**Published:** 2020-05-11

**Authors:** Domenico Iacopetta, Camillo Rosano, Marco Sirignano, Annaluisa Mariconda, Jessica Ceramella, Marco Ponassi, Carmela Saturnino, Maria Stefania Sinicropi, Pasquale Longo

**Affiliations:** 1 Department of Pharmacy, Health and Nutritional Sciences, University of Calabria, Via P. Bucci, 87036 Arcavacata di Rende, Italy; domenico.iacopetta@unical.it (D.I.); s.sinicropi@unical.it (M.S.S.); 2 Biopolymers and Proteomics IRCCS, Ospedale Policlinico San Martino–IST, Largo R. Benzi 10, 16132 Genova, Italy; camillo.rosano@gmail.com (C.R.); ponassi@gmail.com (M.P.); 3 Department of Biology and Chemistry, University of Salerno, Via Giovanni Paolo II, 132, 84084 Fisciano, Italy; msirignano@unisa.it (M.S.); plongo@unisa.it (P.L.); 4 Department of Science, University of Basilicata, Viale dell’Ateneo Lucano 10, 85100 Potenza, Italy; carmela.saturnino@unibas.it

**Keywords:** human topoisomerases I and II, tubulin, women cancers, docking studies, carbene complexes

## Abstract

Herein, we report the synthesis and the multiple anti-tumor properties of new gold and silver carbene complexes. The chemical modifications, grounded on our previous studies, led us to identify a good lead complex, gold-based, whose biological features are very exciting and promising in the anti-cancer research and could be further developed. Indeed, the bis-[4,5-dichloro-(*N*-methyl-*N*’(2-hydroxy-2-phenyl)ethyl-imidazole-2-ylidene)gold(I)]^+^[dichloro-gold]^−^ (**AuL7**) complex possesses the ability to interfere with at least three important and different intracellular targets, namely the human topoisomerases I and II and tubulin, which are able to modulate metabolic processes not directly correlated each other. We proved that the modifications of the ligands structure in **AuL7**, with respect to another already published complex, i.e., bis-[4,5-dichloro-(*N*-methyl-*N*’(cyclopentane-2ol)-imidazole-2-ylidine)gold(I)]^+^[dichloro-gold]^−^ (**AuL4**), produce a different behavior toward tubulin-polymerization process, since **AuL7 is** a tubulin-polymerization inhibitor and **AuL4** a stabilizer, with the final same result of hampering the tumor growth. Taken together, our outcomes designate **AuL7** as a promising compound for the development of multi-targeted anti-cancer therapies.

## 1. Introduction

Tumorigenesis is a complex process involving different signaling pathways. Thus, in some cases, a single-target therapy may exhibit side effects, lack of efficacy, and drug resistance onset. For these reasons, multi-target molecules able to act with a synergistic mechanism could represent a good strategy [1,2]. In recent years, the clinical application of therapies targeting specific proteins or genes, involved in cancer development, have prolonged survival and improved the quality of life of oncologic patients [3]. In particular, women cancers, mostly those affecting reproductive organs and breast, are considered a health alarm for their incidence and for their malignancy; in fact, statistic data report that diagnoses and mortality rate of uterine and breast cancers have increased worldwide in recent years [4,5].

Breast cancer is the primary cause of mortality among women 45–55 years old and is the second leading cause of cancer-induced death. It afflicts inner layer of milk glands or lobules, and ducts [6]. Among the different types of breast cancer, the most aggressive and with the poorer prognosis is the triple-negative one, characterized by the absence of estrogen and progesterone receptors and by the lack of overexpression for human epidermal growth factor receptor 2 (HER2) protein. Consequently, triple-negative breast cancer does not respond to hormonal therapy or HER2 protein receptors drugs. Moreover, a strictly relation between breast and uterine cancers has been demonstrated [7]. Considering all the aspects above-mentioned, there is an urgent need to find new effective agents, lacking of toxic effects and able to act against several important biologic targets involved in DNA metabolism and/or crucial keys in cancer development, such as tubulin and topoisomerases. In this context, Khanna et al. in 2014 reported a new series of Ag(I) and Hg(II)–carbene complexes as good inhibitors of tubulin, an important target involved in crucial cellular roles, such as mitosis, cell signaling, intracellular trafficking, and angiogenesis [8]. Moreover, we have recently reported an overview regarding the use of gold, silver and copper complexes as inhibitors of other important enzymes involved in cancer cells proliferation and in DNA metabolism, namely the human topoisomerases I and II [9].

**Figure 1:**
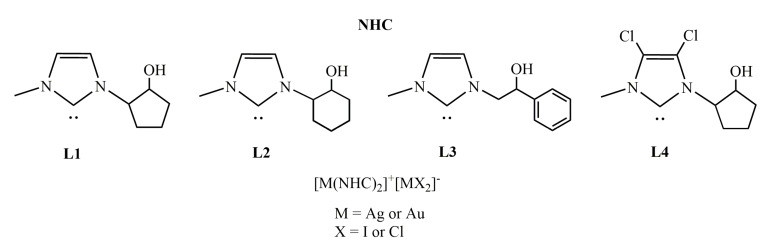
NHC ligands and Ag and Au respective complexes.

This work takes inspiration from the above-mentioned literature data and interesting results recently obtained from some of us [10,11], who synthesized silver and gold complexes with *N*-heterocyclic carbene (NHC) ligands reported in Figure 1, detecting a good anti-tumor activity.

Our previous results highlighted that the structural modifications carried out on the **L1** ligand, i.e., the introduction of the two chlorine atoms on the NHC backbone, produced the ligand **L4**, whose complexes showed a higher anti-tumor activity.

**Figure 2:**
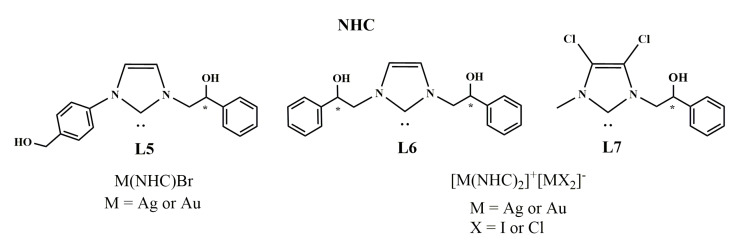
New synthetized Ag and Au complexes.

In this work, we tried to improve the biological activity by modifying part of the structure, starting from the consideration that the nitrogen substituent [(2-hydroxy-2-phenyl) ethyl] may be the most important part of the NHC ligand in the metals-**L3** complexes. Thus, we decided to replace the methyl group linked to nitrogen of imidazole ring with a 2-hydroxy-2-phenyl ethyl or 4-(hydroxymethyl)phenyl group (Figure 2). These substituents could represent a suitable compromise between solubility and lipophilicity; in fact, another alcoholic function would make the complex more soluble in a physiological environment and the aryl group could increase the lipophilia of the complexes. We also synthesized the **L7** ligand, in which the hydrogens on the bridge of the imidazole ring of ligand **L3,** were replaced by two chlorine atoms (see Figure 2). This substitution would further increase the lipophilia and hopefully the biological activity.

On the bases of these considerations, in this paper we report the synthesis and the biological evaluation of four new coinage complexes with *N*-[4-(hydroxymethyl)phenyl]-*N*’-[(2-hydroxy-2-phenyl)ethyl]-imidazole-2-ylidene] (**L5**) or [*N*,*N*’-(2-hydroxy-2-phenyl)ethyl]-imidazole-2-ylidene] (**L6**) as ligands (see Figure 2). Moreover, we studied other two carbene complexes, namely bis-[4,5-dichloro-(*N*-methyl-*N*’(2-hydroxy-2-phenyl)ethyl-imidazole-2-ylidene]silver(I)]^+^[di-iodide-silver]^−^ (**AgL7**) and bis-[4,5-dichloro-(*N*-methyl-*N*’(2-hydroxy-2-phenyl)ethyl-imidazole-2-ylidene]gold(I)]^+^ [dichloro-gold]^−^ (**AuL7**) (see Figure 2), whose synthesis has been recently reported [12].

The anti-tumor activity of all these complexes was evaluated on two breast carcinoma cell lines, MCF-7 and MDA-MB-231, and on two uterine carcinoma cell lines (HeLa and ISHIKAWA). The obtained results showed an interesting anti-tumor activity of the compound **AuL7**, in particular on the highly aggressive and metastatic MDA-MB-231 cell line (IC_50_ = 2.10 ± 0.7 μM). In vitro and in silico studies demonstrated that this compound was able to interfere with the tubulin polymerization and it resulted a valid hTopo I and II inhibitor (already at the concentration of 1 µM). Thus, these results highlighted that **AuL7** can be considered a promising lead agent useful for future investigation that could allow the development of new multi-targets agents in cancer treatment.

## 2. Results

### 2.1. Chemistry

All the syntheses were made using a slightly modified literature procedures [13,14,15,16,17].

#### 2.1.1. Synthesis of *N*-[4-(hydroxymethyl)phenyl]-*N*’-[(2-hydroxy-2-phenyl)ethyl]-imidazolium bromide (**L5**)

**Scheme 1:**
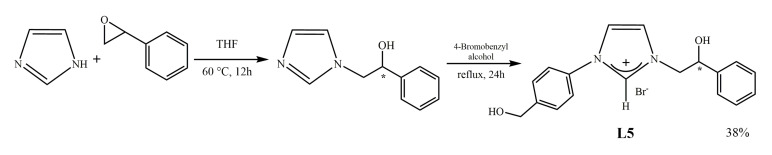
Synthesis of *N*-[4-(hydroxymethyl)phenyl]-*N*’-[(2-hydroxy-2-phenyl)ethyl]-imidazolium bromide (**L5**).

*N*-[4-(hydroxymethyl)phenyl]-*N*’-[(2-hydroxy-2-phenyl)ethyl]-imidazolium bromide (**L5**) was prepared by reaction of imidazole and styrene oxide in tetrahydrofuran (THF). The monoalkylated was obtained after the opening of epoxy-ring. Then, the arylation of the second nitrogen atom using 4-bromo-benzylalcohol to produce the racemic mixture of the salts **L5** (see Scheme 1) was performed.

The synthesized compound was purified following common procedures and isolated in good yield (see experimental part). ^1^H and ^13^C NMR (Nuclear Magnetic Resonance), elemental analysis (C, H, N) and mass spectroscopy allowed us to have an unambiguous structural determination.

The NMR spectra show all the expected signals (see experimental part), in particular the signal of the imidazolium proton on carbon 2 at 8.70 ppm and that of the carbon 2 at 141.6 ppm are to be highlighted.

The elemental analysis gives the predictable composition and ESI-MS spectrum shows the peak leading at 188.1 Dalton attributable to [C_11_H_12_N_2_O]^+^, the pro-ligand after the loss of (hydroxymethyl)phenyl group.

#### 2.1.2. Synthesis of [*N*,*N*’-[(2-hydroxy-2-phenyl)ethyl]-imidazolium iodide (**L6**)

**Scheme 2:**
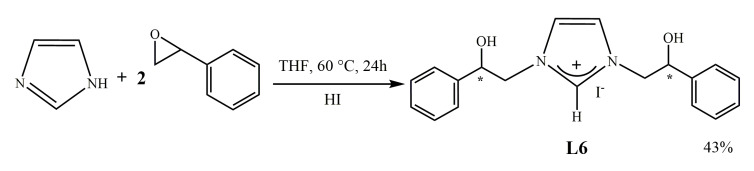
Synthesis of [*N*,*N*’-[(2-hydroxy-2-phenyl)ethyl]-imidazolium iodide (**L6**).

The synthesis of the pro-ligand salt **L6** having on both the nitrogens of the heterocycle the same substituent (2-hydroxy-2-phenyl-ethyl) was carried out as reported in the Scheme 2.

Imidazole is reacted with a double molar amount of 1,2-epoxyethylbenzene using THF as solvent. The reaction mixture is left under stirring for 24 h at 60 °C, then it is filtered, and the soluble fraction is brought to dryness. In this reaction, the opening of the epoxy-ring occurs with formation of the dialkylated product. The salt was purified by dissolution in acetone and the intermediate was obtained by precipitation. Afterward it was dissolved in dichloromethane and HI was added. The whole is left under stirring for about 1 h, at room temperature, and filtered. The salt was characterized by ^1^H and ^13^C NMR spectroscopy, mass spectrometry, and elemental analysis.

The NMR spectra of salt **L6** show all the expected signals and among these of particular relevance is the proton of imidazolium carbon 2 at 9.03 ppm in the ^1^H NMR and the carbon 2 at 141.2 ppm in the ^13^C NMR.

The elemental analysis and ESI-MS that shows the peak leading at 309.1 Dalton attributable to [C_19_H_21_N_2_O_2_]^+^ confirm the hypothesized structure.

#### 2.1.3. Synthesis of *N*-[4-(hydroxymethyl)phenyl]-*N*’-[(2-hydroxy-2-phenyl)ethyl]-imidazole-2-ylidene]-silver(I)bromide] (**AgL5**)

**Scheme 3:**
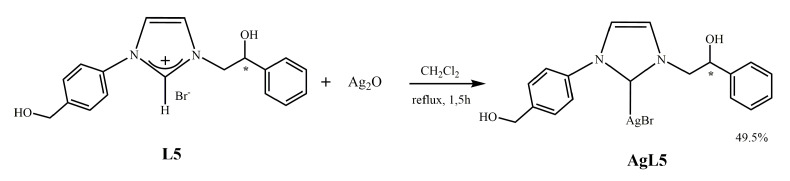
Synthesis of *N*-[4-(hydroxymethyl)phenyl]-*N*’-[(2-hydroxy-2-phenyl)ethyl]-imidazole-2-ylidene]silver(I)bromide] (**AgL5**).

The salt **L5** was reacted in CH_2_Cl_2_ with silver oxide (Ag_2_O) in nitrogen atmosphere and in the presence of molecular sieves. In these conditions, as earlier reported [10], the silver oxide deprotonates the carbon 2 giving the corresponding Ag–NHC complex (**AgL5**) (see Scheme 3). The molecular sieves are used to capture the water, which is a byproduct of the reaction, and to shift the equilibrium to the right. The complex was purified, as reported in the experimental part, and characterized by NMR, elemental analysis, and mass spectroscopy. In the ^1^H NMR spectrum, due to the formation of the carbenic carbon, disappear the proton on carbon 2. The signal for the carbene carbon in NHC Ag(I) complex **AgL5** is invisible. This phenomenon has been reported also for some other Ag(I) carbene complexes, which may result from the fluxional behavior of NHC Ag(I) complexes [18,19,20,21]. ESI-MS spectrum gives a multiplicity of signals between 483.0 and 485.8 Dalton attributable to [C_18_H_18_AgBrN_2_O_2_ + H^+^]^+^, due to two silver (107 and 109) and two bromine (79 and 81) isotopes with a very similar relative abundance.

#### 2.1.4. Synthesis of *N*-[4-(hydroxymethyl)phenyl]-*N*’-(2-hydroxy-2-phenyl)ethyl]-imidazole- 2-ylidene]-gold(I)bromide](**AuL5**)

**Scheme 4:**
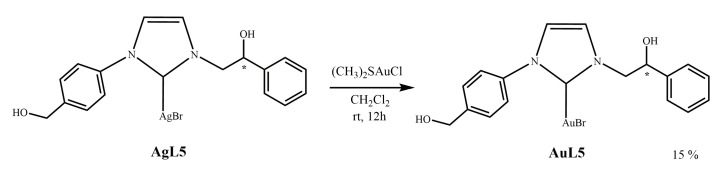
Synthesis of *N*-[4-(hydroxymethyl)phenyl]-*N*’-(2-hydroxy-2-phenyl)ethyl]-imidazole-2-ylidene]-gold(I)bromide](**AuL5**).

The gold complex **AuL5** was obtained by trans-metalation from the corresponding silver complex (see Scheme 4).

The silver complex **AgL5** is reacted with chloro-(dimethylsulfide)-gold(I) [(CH_3_)_2_SAuCl] in dichloromethane. The reaction was left for 1 h at room temperature, then the mixture was filtered, and the solvent removed in vacuo. The obtained solid was characterized by ^1^H NMR and ^13^C NMR, mass spectrometry, and elemental analysis.

The ^1^H and ^13^C spectra of the product show multiple signals for protons and carbons. They are probably due to more species present in solution that are in dynamic equilibrium among them and observable on the NMR time scale. The latter are attributable to species in which the alcoholic oxygen could be coordinated or not to the metal. In ^13^C NMR spectrum of complex **AuL5** the signal for the carbene carbon appears at 161.2 ppm. The success of the synthesis of the gold complex indirectly confirms the formation of the silver precursor from which it derives.

Mass spectroscopy 571.3 and 573.0 Dalton attributable to [C_18_H_18_AuBrN_2_O_2_ + H^+^]^+^ is consistent with NHC-AuBr complex.

#### 2.1.5. Synthesis of bis-[*N*,*N*’-(2-hydroxy-2-phenyl)ethyl]-imidazole-2-ylidene]silver(I)]^+^[di-iodide-silver]^−^ (**AgL6**)

**Scheme 5:**
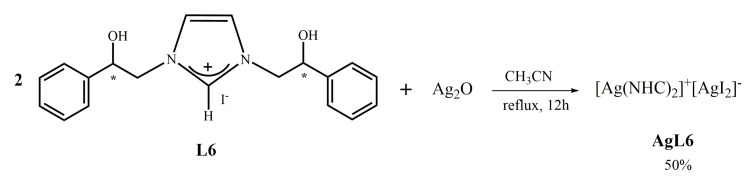
Synthesis of [*N*,*N*’-(2-hydroxy-2-phenyl)ethyl]-imidazole-2-ylidene]-silver(I)]^+^[di-iodide-silver]^−^ (**AgL6**).

This synthesis is carried out as that of complex **AgL5**, but in a different solvent, CH_3_CN instead of CH_2_Cl_2_, for problems related to solubility (see Scheme 5). ^1^H NMR spectrum shows the predictable signals.

As for the complex **AgL5**, also in this case the signal of the carbenic carbon is not visible in the spectrum ^13^C NMR.

The ESI-MS shows peaks at 482.8 and 485.3 Dalton indicative of the fragmentation of the complex with two NHC ligands coordinated with the metallic atom. The observed fragment, corresponding to [C_22_H_22_AgN_4_O_2_ + H^+^]^+^ comes from the complex in which the two ligands lost the group 2-hydroxy-2-phenyl-ethyl.

The elemental analysis suggests a molar ratio between metal, NHC and halogen of 1:1:1, therefore it is plausible to assume the structure [Ag(NHC)_2_]^+^[AgI_2_]^−^. On the other hand, in the literature is reported an Ag complex with two similar NHC coordinated [21,22]. The complex was characterized by ^1^H NMR, ^13^C NMR, mass spectrometry and X-ray analysis [22].

#### 2.1.6. Synthesis of bis-[*N*,*N*’-(2-hydroxy-2-phenyl)ethyl]-imidazole-2-ylidene]gold(I)]^+^[dichloro-gold]^−^ (**AuL6**)

**Scheme 6:**
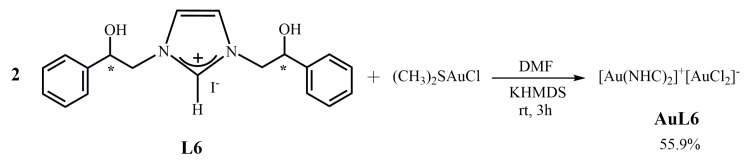
Synthesis of bis[*N*,*N*’-(2-hydroxy-2-phenyl)ethyl]-imidazole-2-ylidene]-gold(I)]^+^[dichloro-gold]^−^ (**AuL6**).

In this synthesis the salt **L6** is reacted in DMF (dimethylformamide) with the base potassium-bis-(trimethylsilyl)-amide and with chloro-(dimethylsulfide)-gold(I) [(CH_3_)_2_SAuCl] (see Scheme 6). The reaction is left under stirring for 3 h at room temperature. The obtained complex was characterized by ^1^H and ^13^C NMR and mass spectrometry.

ESI-MS(CH_3_CN, *m*/*z*): 669.0 Dalton attributable to [C_32_H_31_AuN_4_ + H^+^]^+^.

As in the case of the silver complex, also for the gold complex the mass suggests a gold ion with two coordinated ligands, which lost the four alcohol groups and a phenyl group and since the elemental analysis gives a molar ratio among gold, ligand and chloride of 1:1:1, it is possible to hypothesize a structure consisting of [(NHC)_2_Au]^+^ cation and of [AuCl_2_]^−^ anion. It is worth noting that we have not been able to obtain a solid state structure by X-ray analysis, because despite the many attempts made (dissolution in various solvent media and slow decrease of temperature, various solvent-non-solvent mixtures, etc.) we were unable to obtain suitable crystals.

The hypothesized structure can be stabilized by means of possible intramolecular hydrogen bonds.

To correlate the coordination stability of the synthesized compounds with their evaluated cytotoxic activities, hydrolysis tests were performed. Probably, some biologically inactive species could be obtained by the rapid hydrolysis of these complexes, whereas an active species, if the NHC ligand remain coordinated to the metal, could be achieved. The hydrolysis stability of new silver and gold complexes was determined in 10% DMSO-d_6_ aqueous solution, by ^1^HNMR analysis. The complexes show high hydrolytic stability; in fact, all of them are hydrolyzed for less than 5% after 24 h. These data provide sufficient evidence that the presence of NHC coordinating groups on the metals are effective for the stabilization of the complexes.

### 2.2. Biology

#### 2.2.1. Anti-Tumor Activity

The anti-tumor activity of these complexes was evaluated on two breast carcinoma cell lines, MCF-7 and MDA-MB-231, and on two uterine carcinoma cell lines (HeLa and ISHIKAWA).

The obtained results (Table 1), established an interesting anti-tumor activity for the compound **AuL7**, in particular against the highly aggressive and metastatic MDA-MB-231 cell line (IC_50_ = 2.10 ± 0.7 μM). The corresponding silver complex **AgL7** exhibited a good anti-tumor activity on this cell line (IC_50_ = 3.22 ± 1.2 μM) and, as well, toward the uterine cell lines (IC_50_ values = 9.76 ± 0.6 and 6.55 ± 0.9 μM on HeLa and ISHIKAWA, respectively). The good anti-tumor activity of **L7** complexes could be related to the presence of chlorine atoms on the imidazole nucleus. In fact, we found that the gold analog **AuL3,** without the chlorine atoms on the NHC backbone, was less active against the MDA-MB-231 cell lines (IC_50_ = 3.43 ± 1.0 μM) and the silver complex **AgL3** was completely inactive. Gold complexes containing the **L5** and **L6** ligands were also able to inhibit the cells proliferation, but lesser than **L7** series. Instead, **AgL5** and **AgL6** possessed a very low activity toward the cell lines used (see Table 1). The latter outcomes underline, once again, that the halogen atoms are important for the anti-tumor activity. *Cis*platin, used as reference molecule, exhibited a strong anti-tumor activity toward the cancer cells, together with a high toxicity against normal human mammary epithelial MCF-10A and human embryonic kidney epithelial Hek-293 cells (81.3 ± 1.2 and 16.8 ± 0.4 μM, respectively). All the new synthetized complexes, instead, were found to possess no or low cytotoxicity on the normal cell lines (see Table 1). In particular, the most active compound **AuL7** was inactive toward the MCF10A cells, but quite cytotoxic, if compared to the other compounds, toward the human embryonic kidney epithelial Hek-293 cell lines with an IC_50_ value of 65.04 ± 0.7 μM. Several literature works report that Quercetin plays a protective role against *Cis*platin-induced nephrotoxicity [23,24,25]. The mechanism underlying this toxicity is not yet clearly understood, but recent in vitro and in vivo studies indicate that it is closely associated with lipid peroxidation [24,26] and the oxygen free radicals (ROS) production [24,27]. The oxidative stress responsible for *Cis*platin-induced nephrotoxicity can be reduced by using different antioxidants, as Quercetin that is a powerful ROS scavenger, a metal chelator and an inhibitor of the xanthine oxidase in in vitro and in vivo systems [28]. Moreover, Quercetin shows, even at low concentrations, a high radical scavenging activity and the highest antioxidant potential if compared to vitamin C and trolox (known antioxidants used as control) and to other common powerful antioxidants (Resveratrol, Ferulic Acid, Gallic Acid, Kampferol, etc.) [29,30]. On these bases, we evaluated the protective effect of Quercetin against **AuL7**-induced nephrotoxicity on Hek-293 cells. The obtained results show that the pre-treatment for 4 h with Quercetin (at 50 μM) produced an increase of the Hek-293 cells viability of about 1.5 fold (IC_50_ value of 96.90 ± 1.1 μM)compared to those treated with **AuL7** alone (IC_50_ value of 65.04 ± 0.7 μM). The protective role of Quercetin against the **AuL7** cytotoxicity against the renal Hek-293 cell lines represents an interesting starting point for next studies.

#### 2.2.2. Docking Studies

**Figure 3:**
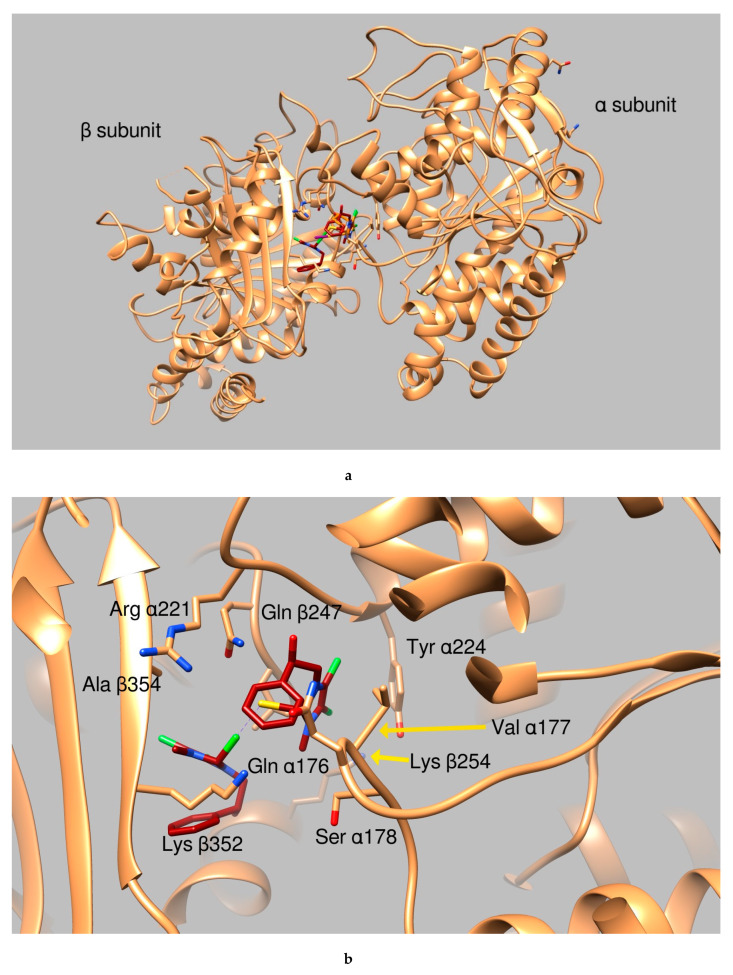

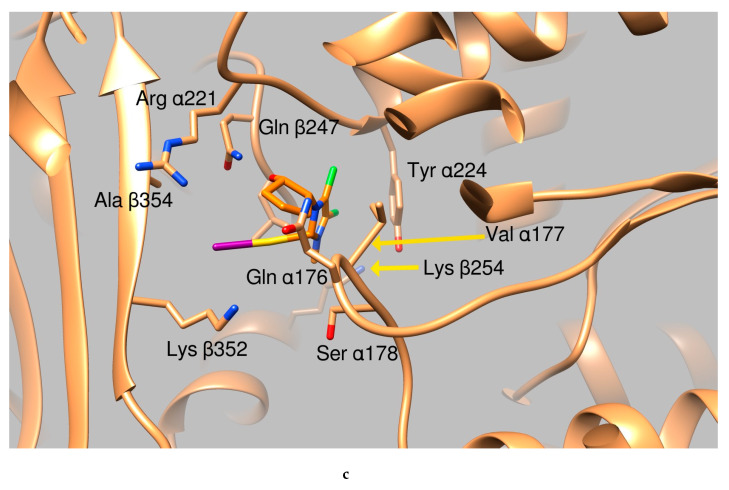
Graphical representation of **AuL4** and **AuL7** binding to the tubulin α:β interface. (**a**) Ribbon representation of the protein dimeric interface (tanned ribbons), bound ligands are reported as sticks. Residues involved in the binding are properly labeled. (**b**) Tubulin dimeric interface in complex with **AuL7** (protein is represented in tanned ribbons, amino acids involved in ligand binding are properly labeled, **AuL7** is drawn as purple sticks). (**c**) Close up of **AuL4** (orange sticks) binding.

To evaluate the binding modes and affinities between our class of newly synthesized compounds and the target proteins, we performed molecular docking simulations using as ligands the molecules described in Figure 3. Using a “blind-docking approach” (no “a priori” information about the binding site was provided to the system), we aimed to identify the most promising candidates and evaluate their binding energies to the three proteins, namely the human topoisomerases I and II and tubulin. In addition, the examination of the binding mode of our compounds with these biological targets could be useful for the discovery and the design of new further molecules with higher affinity for the studied proteins. In these studies, we used all the complexes belonging to the new synthesized series and the already published **AuL4** [11], which was the most active of the previous series.

Considering the results from the simulations, shown in Table 2, we initially considered to be “good candidates” the two compounds **AuL4** and **AuL7**. We based our choice on the compound binding affinity to the different proteins, as calculated by the program Autodock (The program Autodock calculates a binding affinity constant K_i_ based on the binding energy, according to the expression Ki = exp (deltaG/ (R*T)) and on the clusterization of the results, as discussed in previous works [31,32,33]. Finally, the structural pose was examined to evaluate the quality of the protein:ligand interactions. Analyzing further the possible interactions of the two moieties with the residues at the interface between the two tubulin subunits, we notice that **AuL4** forms hydrogen bonds with residues Gln176, Ser 178, and Tyr224 belonging to subunit alpha and with Gln247 and Lys254 of subunit beta. A halogen bond is also formed by the Iodine atom and Lys352 of beta subunit. Summing up, **AuL4** seems to stabilize the contacts between the two subunits, in a way similar to taxol. Contrarily, **AuL7** seems to break this interaction by the insertion of two benzenic rings between residues belonging to the two subunits that normally exchange stabilizing hydrogen bonds. This mechanism is more similar to the vincristine mode of action.

Concerning the simulations using as a target the structures of Topoisomerase I and II, we did not find a good clusterization of the results for any ligand but **AuL7** tested which, according to our method, indicates that there is very low probability of binding for our compounds. On the other hand, **AuL7** binds to topoisomerase I by interaction with residues Arg200, Arg324, Asn327, Lys368, and Asp369 occupying in part the channel where the DNA is normally bound.

Table 2 reports the Binding Energies and Binding constants for each compound tested in silico. The color codes represent the result of the analysis of clusterization and of the binding modes. Particularly:

- green background: good clusterization of the simulation results and ligand binding modes that occur in a protein area which is either involved in DNA binding or in protein-protein interactions (i.e., tubulin oligomer formation, topoisomerase dimerization etc.)
- orange background: no (or small) clusterization of the results indicating low probability of ligand binding
- yellow background: good clusterization of the simulation results but the ligand binding mode occurs in an area not involved in interactions with DNA or in a zone not involved in oligomeric formation.

#### 2.2.3. **AuL7** Induces Cell Cytoskeleton Destabilization

Microtubules are vital for many cellular functions and their damage alters the function of the spindle apparatus, triggering apoptosis in cancer cells [34]. Several known drugs, currently used in cancer treatment, can interfere with the microtubules dynamics. In particular, Vinca alkaloids such as vinblastine are classified as destabilizing agents because they cause microtubule depolymerization whereas taxanes, such as paclitaxel, are able to bind the interior surface of microtubules, inducing stabilization of microtubules and increasing tubulin polymerization [35]. The predictive docking studies suggested that **AuL7** and **AuL4** could interfere with tubulin polymerization in the same manner than vinblastine and paclitaxel, respectively. To investigate whether our compounds act as microtubules-stabilizing or -destabilizing agents, we performed both the in vitro tubulin polymerization and immunofluorescence assays on MDA-MB-231 (**AuL7**) and HeLa cells (**AuL4**).

First, the in vitro tubulin-polymerization inhibition assay is a valid and fast test for studying the ability of our compounds to inhibit or not the tubulin-polymerization reaction by measuring the turbidity variation at 350 nm. In this test, we used an only-vehicle (DMSO) control, paclitaxel and vinblastine, as reference molecules and the compounds **AuL7** and **AuL4**, at 10 µM concentration.

**Figure 4:**
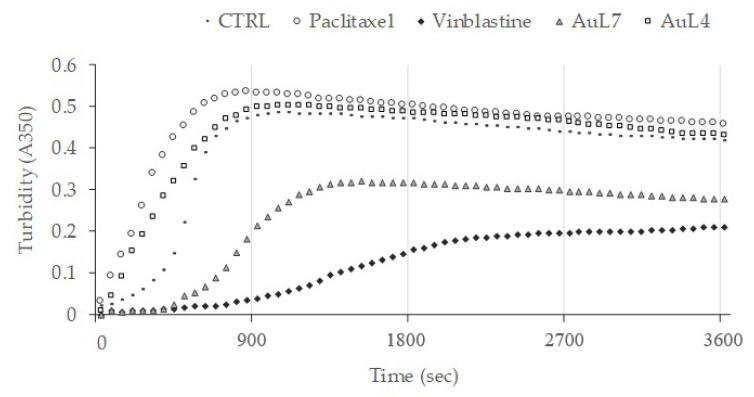
Tubulin-polymerization assay. The effect of compounds **AuL4** and **AuL7** on in vitro tubulin polymerization was evaluated. Two positive control molecules, Paclitaxel and Vinblastine, were used as tubulin-stabilizing and tubulin-destabilizing agents, respectively. DMSO was used as a negative control. Assembly of tubulin into microtubules was determined by measuring the turbidity at 350 nm for 3600 s at 37 °C. All the compounds were used at a concentration of 10 μM.

Results depicted in Figure 4 show a classic polymerization curve for the control reaction (vehicle, DMSO), indeed the increase of turbidity in a time-dependent manner indicates the tubulin heterodimers self-assembling. This reaction reaches the final optical density value at 350 nm (OD_350_) of about 0.43. The curve pertinent to the microtubule-stabilizing agent paclitaxel has a similar trend, but reaches a higher OD_350_ value of about 0.51 and a faster plateau, which means a higher amount and rate of tubulin heterodimers assembly. As expected, vinblastine curve reaches the steady state later (in about 40 min) and with a final turbidity value less than the half of the vehicle reaction, which indicates the tubulin-polymerization inhibition. Surprisingly, a different behavior can been noticed with respect to **AuL7** and **AuL4**; indeed, the exposure of tubulin to **AuL7** seems to produce the reaction inhibition, as indicated by the polymerization curve similar to that of the vinblastine (even though the latter produced a faster and higher tubulin-polymerization inhibition), with a final OD_350_ value of about 0.25. Conversely, **AuL4** seems not to hamper the tubulin-polymerization reaction, since its curve is between the curves of the vehicle and paclitaxel reactions, respectively. Our outcomes clearly indicates that **AuL7** is a tubulin-polymerization inhibitor, whereas **AuL4** is not, even though its initial curve trend, the final OD_350_ value, slightly higher than that of DMSO reaction, together with docking hypotheses, brought us to suspect a stabilizing role in tubulin assembly.

#### 2.2.4. Immunostaining Studies

With the aim to deepen the role of **AuL7** and **AuL4** in tubulin-polymerization reaction and, thus, how they could affect the cytoskeleton dynamics, we performed immunostaining studies using the MDA-MB-231 and HeLa cells exposed, respectively, to **AuL7** and **AuL4**.

**Figure 5:**
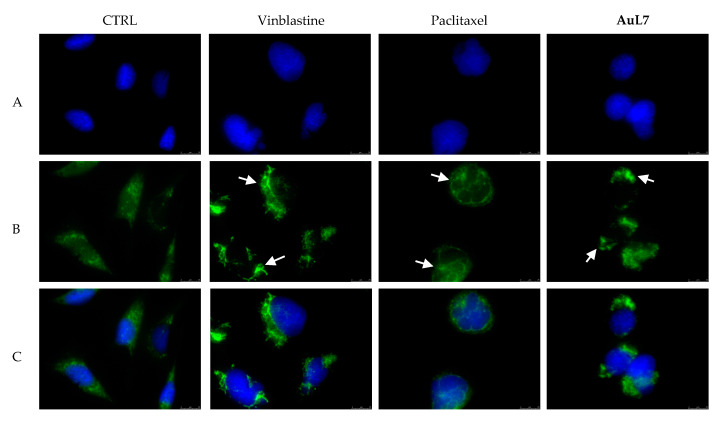
Immunofluorescence studies. MDA-MB-231 cells were treated with **AuL7**, Vinblastine, Paclitaxel (used at their IC_50_ values) or with a vehicle (CTRL) for 24 h. After treatment, the cells were methanol fixed, incubated with primary and secondary antibodies, stained with 4′,6-diamidino-2-phenylindole (DAPI) and observed and imaged under the inverted fluorescence microscope at 63× magnification (see Materials and methods). Vehicle-treated cells (CTRL) exhibited a normal arrangement and organization of the cytoskeleton. Microtubules of MDA-MB-231 cells treated with vinblastine and paclitaxel showed an irregular arrangement and organization: particularly, tubulin crystal formation (white arrows, panel **B**, vinblastine) and tubulin bundles and thicken fibers (white arrows, panel **B**, paclitaxel) can be noted for vinblastine and paclitaxel respectively. Treatment with **AuL7** shows a vinblastine-like mode of action (white arrows, panels **B**, **AuL7**). Panels **A**: DAPI, excitation/emission wavelength 350 nm/460 nm; panels **B**: β-tubulin (Alexa Fluor^®^ 488) excitation/emission wavelength 490 nm/515 nm; panels **C** show a merge. Representative fields are shown.

**Figure 6:**
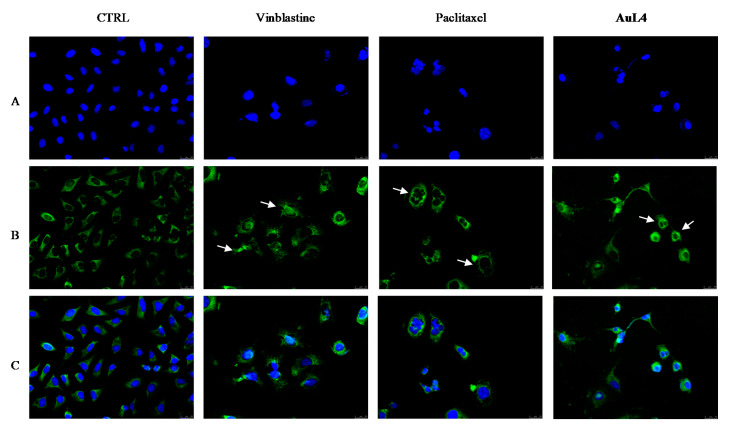
Immunofluorescence studies. HeLa cells were treated with **AuL4**, Vinblastine, Paclitaxel (used at their IC_50_ values) or with a vehicle (CTRL) for 24 h. After treatment, the cells were methanol fixed, incubated with primary and secondary antibodies, stained with DAPI and observed and imaged under the inverted fluorescence microscope at 20× magnification (see Materials and methods). Vehicle-treated cells (CTRL) exhibited a normal arrangement and organization of the cytoskeleton. Microtubules of HeLa cells treated with vinblastine and paclitaxel showed an irregular arrangement and organization: particularly, tubulin crystal formation (white arrows, panel **B**, vinblastine) and tubulin bundles and thicken fibers (white arrows, panel B, paclitaxel) can be noted for vinblastine and paclitaxel respectively. Treatment with **AuL4** shows a paclitaxel-like mode of action (white arrows, panels B, **AuL4**). Panels **A**: DAPI, excitation/emission wavelength 350 nm/460 nm; panels **B**: β-tubulin (Alexa Fluor^®^ 488) excitation/emission wavelength 490 nm/515 nm; panels **C** show a merge. Representative fields are shown.

As shown in Figure 5 and Figure 6 (panels B, CTRL), the vehicle-treated cells exhibited a regular organization of the microtubule network, indeed the filiform microtubules are distributed regularly into the cytoplasm of both cell lines used in this experiments. The exposure of cells to vinblastine produced, as expected, the microtubules disorganization (Figure 5 and Figure 6, panels B), with microtubules that become thicker around the cell nuclei and accumulate in punctiform structures (crystals, indicated by white arrows). In contrast, paclitaxel, a well-known microtubule-stabilizing agent, induces the microtubules non-reversible polymerization in both cell lines, with a higher formation of tubulin bundles and thicken fibers (Figure 5 and Figure 6, panels B). Moreover, the presence of multinucleated cells with condensed nuclei (see white arrows), due to the engagement of the spindle checkpoint and the mitotic arrest, suggests that apoptosis could be taking place. In this scenario, **AuL7** and **AuL4** exhibit a contrasting behavior: indeed, **AuL7** has a vinblastine-like mode of action, as visible in Figure 5 panel B. Tubulin distribution in the cell cytoplasm appears unfair and is tightly packed around cells nuclei (see white arrows) with a higher presence of tubulin crystals. Conversely, **AuL4** treatment provokes the disruption of the normal microtubule architecture with a higher presence of round cells with multiple micronuclei or multilobed nuclei (Figure 6, panel B, white arrows), indicating a paclitaxel-like behavior. These deductions agree with the previous experiments, indicating doubtless that **AuL7** is a tubulin-polymerization inhibitor whereas **AuL4** is a microtubule-stabilizing agent.

#### 2.2.5. Human Topoisomerases Inhibition

**Figure 7:**
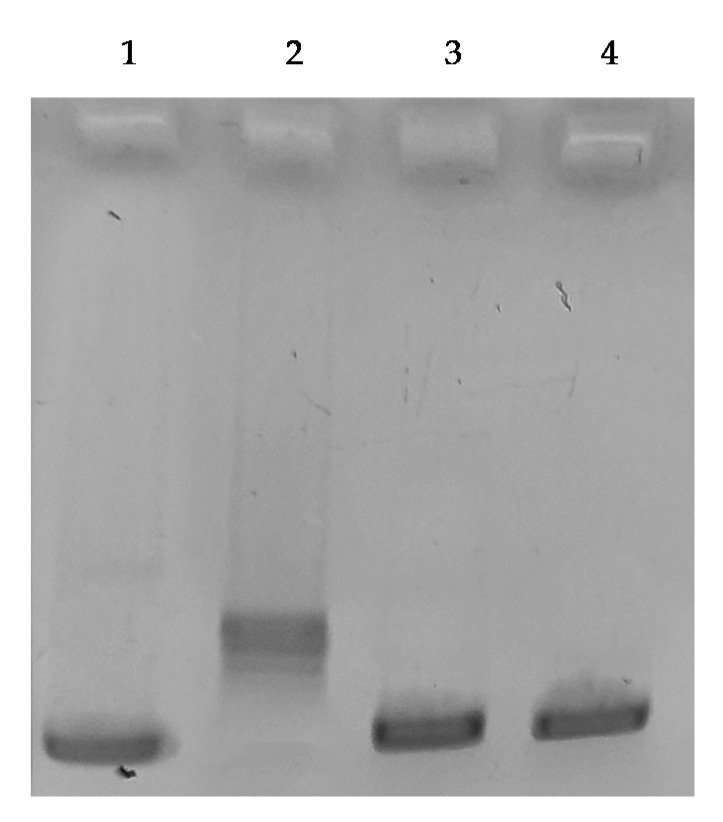
hTopoI assay. Supercoiled DNA was incubated without or with human Topoisomerase I in the absence or presence of the test compounds at 1 μM: lane 1, plasmid pHOT1, lane 2, vehicle (DMSO), lane 3, **AuL7** 1 μM and lane 4, **AuL4** 1 μM.

Topoisomerases are essential enzymes involved in DNA metabolism, because of their ability to cause temporary DNA strand breakage, and tightly involved, if overexpressed, in cancer development. Anti-cancer agents targeting one or both topoisomerases isoforms are successfully used in cancer treatment [2,9,28,36,37,38,39] and, more recently, many studies have demonstrated that metal complexes are good inhibitors. To assess the capability of **AuL7** and **AuL4** to inhibit human topoisomerases, we performed an in vitro assay for human topoisomerase I (hTopo I) activity, based on the relaxation of supercoiled DNA, and an assay for human topoisomerase II (hTopo II), based on the decatenation of double-stranded DNA. Figure 7 shows the totally inhibition of the hTopo I supercoil relaxing activity in the presence of compounds **AuL7** and **AuL4** at the concentration of 1 μM (Figure 7, lanes 3 and 4 respectively), as demonstrated by a clear band of the supercoiled plasmid pHOT1 DNA at the bottom of the gel. The only-vehicle (DMSO) control experiment (Figure 7, lane 2), shows the full hTopo I activity, under the same experimental conditions, which produces the canonical DNA fragments pattern.

**Figure 8:**
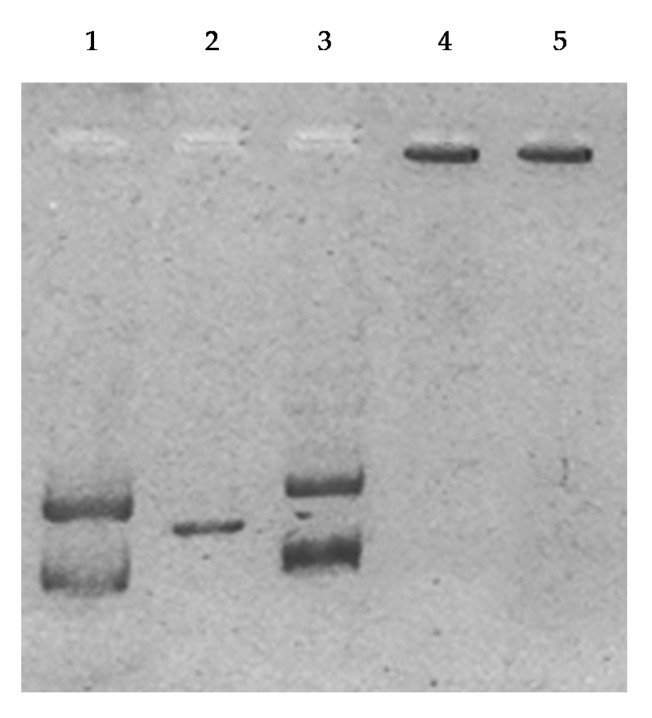
hTopo II inhibition assay. kinetoplast DNA (kDNA) was incubated with human topoisomerase II in the absence (lane 3, vehicle DMSO) or presence of compound **AuL7** and **AuL4** at1μM (lane 4 and 5 respectively). Lane 1 and 2, decatenated and linear kDNA, respectively.

Next, we performed the hTopo II assay using interlocked kinetoplast DNA (kDNA) as substrate and compounds **AuL7** and **AuL4** at 1 μM concentration. As shown in Figure 8, lane 3, the control reaction (DMSO only) shows two bands related to the decatenation products, indicating the full hTopoII activity. The exposure of hTopoII to the tested compounds produces a total blockade of hTopoII activity, as demonstrated by the presence of one single DNA band at the top of gel that represents the kinetoplast DNA (kDNA) (Figure 8, lanes 4 and 5). Decatenated and linear kDNA (Figure 8, lanes 1 and 2) are used as markers.

#### 2.2.6. **AuL7** Induces Cancer Cells Apoptosis

**Figure 9:**
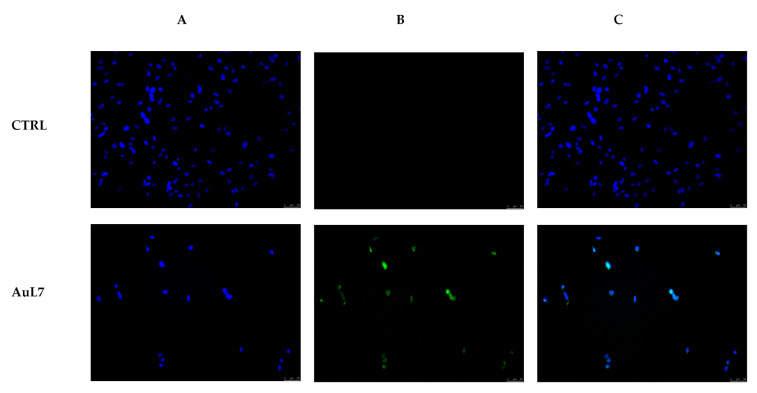
MDA-MB-231 cells were treated with **AuL7** at a concentration corresponding to its IC_50_ value or with the vehicle (CTRL) for 24 h. Processed cells were observed under an inverted fluorescence microscope at 20× magnification. The green nuclear fluorescence, in **AuL7**-treated cells, indicates apoptotic death. Panels **A**: DAPI (CTRL and **AuL7**) excitation/emission wavelength 350 nm/460 nm. Panels **B**: CF™488A (CTRL and **AuL7**) excitation/emission wavelength 490 nm/515 nm. Panels **C**: a merge of panels A and B. Fields are representative of three separate experiments.

Once demonstrated that **AuL7** possesses a multi-targeted activity, namely towards tubulin and both human topoisomerases, we performed the terminal deoxynucleotidyl transferase dUTP Nick-End Labeling (TUNEL) assay in MDA-MB-231 cell lines. Cells were exposed to **AuL7** at its IC_50_ value for 24 h, then processed as described in the experimental section. The obtained results clearly show a green nuclear fluorescence visible only in **AuL7**–treated cells (Figure 9, panel B) and not in the vehicle-treated cells (CTRL, Figure 9, panel B). Overlay channel (**AuL7** and CTRL, Figure 9, panel C) is also shown. Our outcomes indicate that the blockade of tubulin polymerization and human topoisomerases leads to MDA-MB-231 cells death by apoptosis.

#### 2.2.7. **AuL7** Induces Reactive Oxygen Species Formation and the Mitochondria-Mediated Apoptosis Pathway

The DNA damage induced by the blockade of hTopos and the perturbation of the cytoskeleton structure due to its involvement in microtubule formation, are cellular events strictly connected with cell death via the apoptotic pathway. Moreover, the interference with the above-mentioned events may lead to a rise in intracellular Reactive Oxygen Species (ROS) production, another important process tightly related to the apoptosis induction under both physiological and pathological conditions. Previous studies have shown that oxidative stress can cause cellular apoptosis via intrinsic mitochondrial cell death pathway [40,41].

Moreover, ROS generation after treatment of cancer cells with gold-based compounds is already described in the literature [11,42,43,44,45]. In particular, it was reported that gold-NHC complexes were able to trigger the mitochondrial death pathway, producing a modification of the Bax/Bcl-2 ratio leading to an increase of the cytosolic cytochrome c and to a decrease of pro-caspase 9 and 3 [42,43]. Moreover, other gold complexes increased ROS generation, reducing thioredoxin reductase and proteasome activities, essential components that regulates cellular redox status [44,45]. Recently, we have highlighted that gold-NHC complexes played a key role in the mitochondria disruption and cytochrome c release from its natural site, which induced the activation of the intrinsic apoptotic pathway in a ROS-dependent manner [11].

**Figure 10:**
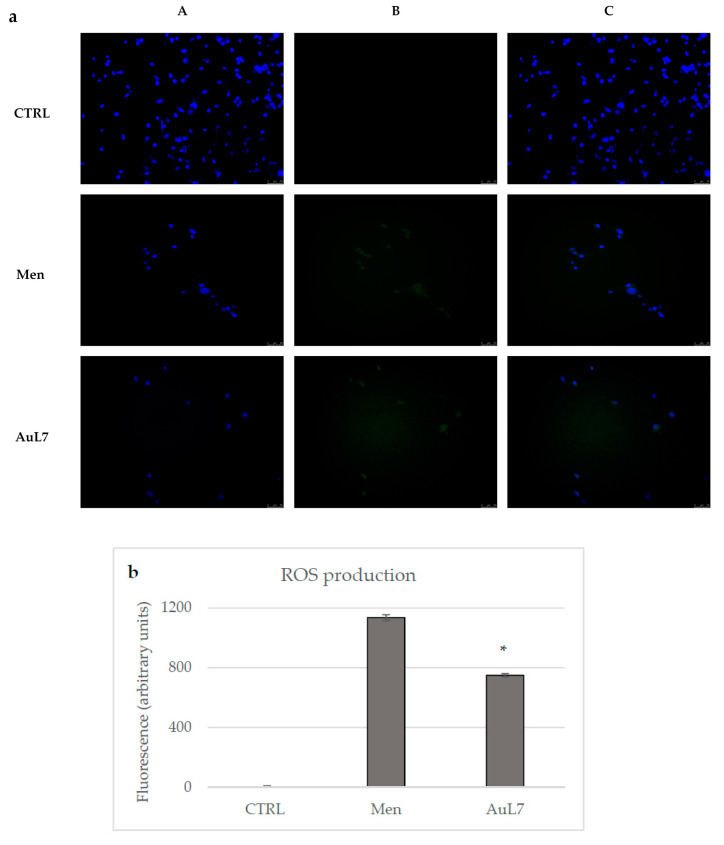
ROS induction by **AuL7** treatment in MDA-MB-231 cells. (**a**) ROS production was evaluated by using DCF-DA in MDA-MB-231 cells treated with Menadione (Men, used as positive control), **AuL7**, or vehicle. Images were acquired at 20× magnification. (**A**): Nuclear stain with DAPI (excitation/emission wavelength 350 nm/460 nm) in treated (Men or **AuL7**) and untreated cells (vehicle, CTRL); (**B**): DCF (excitation/emission wavelength 490 nm/515 nm for CFQ488A) in treated (Men or **AuL7**) and untreated cells (vehicle, CTRL); (**C**): overlay channel; (**b**) Fluorescence quantification. *p < 0.001, **AuL7** vs. Men. Fields are representative of three separate experiments.

With this in mind, the generation of ROS in MDA-MB-231 cells was monitored during the treatment with the most active compound **AuL7** or with menadione, used as positive control. We used 2,7-dichlorofluorescin diacetate (DCF-DA) as a fluorometric probe for ROS detection and their production was examined under an inverted fluorescence microscope, as already reported [11,46]. As shown in Figure 10, the green fluorescence indicative of increased ROS production is present in the cytoplasm of cells treated with menadione (Panel B, Men), and in **AuL7**-treated cells (Panel B, **AuL7**), even though in a lesser extent with respect to the Men-treated cells but significantly higher with respect to the vehicle-treated cells (Panel A, CTRL), used as control. The increase in ROS generation in treated cells was quantified by ImageJ and the results (shown in Figure 10, panel B) indicate that the amount in ROS production in **AuL7**-treated cells is only 1.5-fold lesser compared to the Men-treated cells.

Our data indicate that **AuL7** can increase the oxidative stress in MDA-MB-231 cells, an event related to the above-observed apoptosis.

Next, we investigated whether the observed ROS increase is associated with the apoptosis via the mitochondrial pathway, inducing the mitochondria-to-cytosol translocation of cytochrome *c* and the activation of the caspases cascade [47].

**Figure 11:**
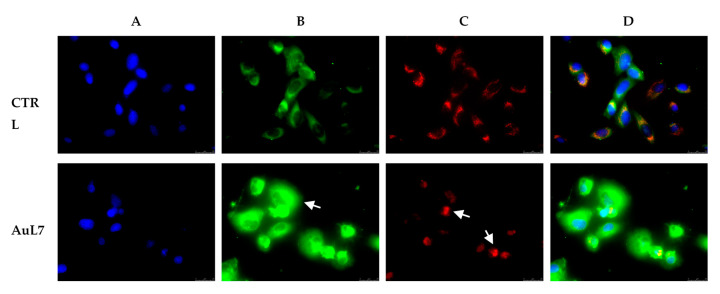
Mitochondria staining and cytochrome c translocation in MDA-MB-231 cells. In vehicle-treated cells, cytochrome c (panel B, CTRL) is localized within intact mitochondria (panel C, CTRL), as visible in panel D (CTRL) where the overlay channel is shown. Treatment with **AuL7** at a concentration corresponding to its IC_50_ value for 24 h induces cytochrome c release in the cytosol (panel B, **AuL7**, white arrow) and loss of mitochondria integrity (panel C, **AuL7**, white arrows). (**A**) nuclear stain with DAPI (excitation/emission wavelength 350 nm/460 nm); (**B**) Alexa Fluor CF 488 (excitation/emission wavelength 490 nm/515 nm); (**C**) MitoTracker Deep Red FM probe (excitation/emission wavelength 644 nm/665 nm); (**D**) Overlay channels. Images were acquired at 63x magnification and representative fields are shown.

At this purpose, we performed immunostaining studies using the mitochondrial probe MitoTracker Deep Red FM and an anti-cytochrome c antibody, to follow the possible release of this protein from the mitochondrial district to the cytoplasm. In DMSO-treated cells the mitochondria were intact (Figure 11, panel C, red fluorescence) and cytochrome c resides in the right place (Figure 11, panel B, green fluorescence), as indicated by the perfect overlay (Figure 11, panel D) of red fluorescence (MitoTracker deep Red FM) with the green one (i.e., the anti-cytochrome c antibody). Instead, the **AuL7**-treated cells showed a loss of mitochondrial structural integrity, as demonstrated by the red fluorescence that accumulates as dotted structures around the cells nuclei (Figure 11, panel C, white arrows). The loss of mitochondrial integrity leads to the cytochrome c release and diffusion into the cytoplasmic compartment, as visible by the increased and delocalized green fluorescence (Figure 11, panel B, white arrow) and by the altered overlay (Figure 11, panel D).

**Figure 12:**
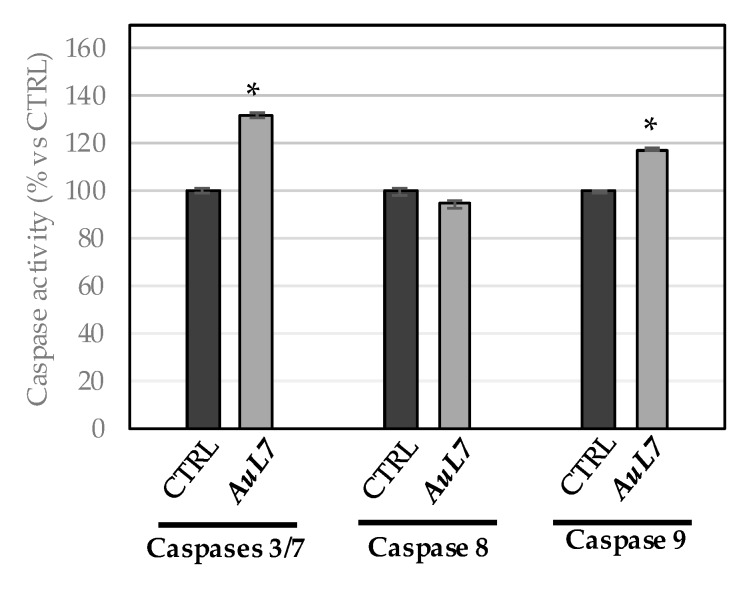
Caspases activity. Activation of caspases 3/7 and 9 due to the treatment of MDA-MB-231 cells with the compound **AuL7** at a concentration equal to 5 μM for 24 h. Columns mean, bars standard deviations (SD), * *p* < 0.001. Results are representative of three separate experiments.

Cytochrome c release from mitochondria induces a series of biochemical reactions that result in caspases activation, a subfamily of cysteine-proteases involved in the initiation of several proteolytic events. In fact, in the cytosolic cytochrome c binds to Apaf-1, which in turn promotes the assembly of a multiprotein complex, namely the “apoptosome”, and the activation of the initiator caspases 8 and 9. These two caspases, in turn cause consequent cleavage of the effectors caspases 3 and 7 [47,48]. With this in mind, we evaluated whether **AuL7** treatment (5 μM) could activate the caspases activity. As shown in Figure 12, the caspase assay demonstrated a discrete increase of caspase-9 activity in MDA-MB-231 cells, whereas the caspase-8 activity resulted unchanged with respect to the control reaction. Additionally, a clear increase of caspases 3/7 activity was also demonstrated, because of the cleavage activity of the initiator caspase-9. Thus, the exposure of MDA-MB-231 cells to **AuL7** induces the intrinsic ROS-mediated apoptotic pathway.

#### 2.2.8. Cell Cycle Assay

**Figure 13:**
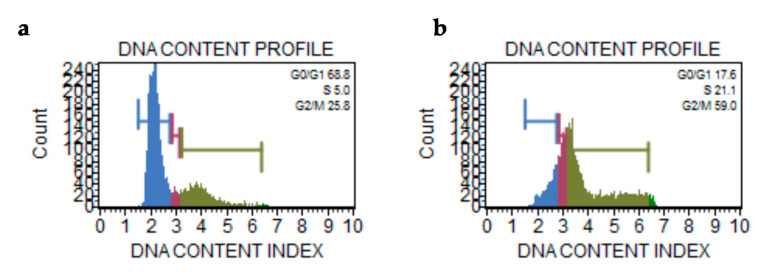
Cycle assay. (**a**) DNA content histogram of MDA-MB-231 cells treated with only DMSO; (**b**) DNA content histogram of MDA-MB-231 cells treated with **AuL7** at the concentration corresponding to its IC_50_ value for 72 h.

It is widely assumed that G2/M checkpoint forbids cells carrying DNA damage or cytoskeleton dysfunctions to undergo mitosis [49]. Therefore, to verify if the **AuL7** treatment can cause modifications in the cell cycle profile, we performed a Muse cell cycle analysis. The results clearly show that the **AuL7** treatment causes an increase of the percentage of MDA-MB-231 cells in G2/M and S phases respectively of 33.2% and 16.1%, compared to MDA-MB-231 control cells (DMSO-treated) (Figure 13). These outcomes strength the already established role of **AuL7** to block both hTopos and tubulin-polymerization reaction.

## 3. Discussion

Presently, there is a general agreement that compounds able to interact simultaneously with different targets might be more active than a single-target agent. Multi-target drugs, used in combination or in sequential order, might be more efficient to block tumor progression, considering the involvement of several, and often unrelated, signaling pathways in cancer development, and might combat the frequent phenomenon of intrinsic and acquired resistance to anti-cancer drugs [50,51,52]. Indeed, under the pressure of a single-targeted drug perturbation, cancer may adopt auxiliary or alternative molecular pathways to survive and produce a more aggressive phenotype, often with metastasization events [53]. Thus, the design and study of new multi-target anti-cancer agents, able to overcome these limitations, represent an exciting challenge for the discovery of next-generation drugs. In this context, microtubules and topoisomerases represent important anti-cancer targets and the combination of both inhibitors, gifted of synergistic effects and low toxicity, should enhance the final therapeutic outcome. In this work, we reported the synthesis and the molecular mechanism evaluation of a new series of metal complexes, with improved biological properties. We also compared the most active metal complex **AuL7** with a previous published one (**AuL4**) and study in deep their peculiar biological features. These new complexes were screened for their anti-tumor properties using two breast and two uterine cancer cell lines, finding out that **AuL7** and **AgL7** were the most active of the series and, particularly, **AuL7** that was the most active against the triple-negative and most aggressive MDA-MB-231 breast cancer cells. The parallel experiment using two different types of normal cell lines demonstrated the total lack of cytotoxicity, with the exception for **AuL7** that showed a certain cytotoxicity toward the renal Hek-293 cells. Literature data report a nephrotoxicity for some metal-based drugs and the protective effect of powerful antioxidants; effectively, the sequential treatment of Quercetin and **AuL7** onHek-293 cells produced a decrease in **AuL7**-induced cytotoxicity, an interesting aspect that will be useful for next studies. In the last few years, the interest of scientists for chemical properties of the gold complexes has increased, because of their interactions with several intracellular targets involved in the anti-proliferative effects, particularly capable of inhibiting both topoisomerase isoforms, whose blockage lead to the programmed cell death [54,55,56,57,58,59]. Among these works, Yan et al. in 2010 [60] published a series of gold complexes with NHC ligands exhibiting in vitro and in vivo interesting biological properties. The best active complex caused the inhibition of DNA relaxation at 10 μM, and, as well, the stimulation of DNA cleavage by TopoI in cervical epithelioid carcinoma cells.

Moreover, several scientific studies demonstrated that metal complexes could interfere the tubulin polymerization, for instance recent advances showed that Ru-, Pt-, Ir-, Ag- and Hg-based complexes are good tubulin-polymerization inhibitors [8,61,62,63]. On the contrary, no scientific evidence has demonstrated the ability of gold metal complexes to interfere with this important target.

This evidence prompted us to investigate the cellular effects of **AuL7** on the human topoisomerases I and II and tubulin, vital for cell metabolism and dramatically involved in cancer onset and progression. In silico studies demonstrated that **AuL7** could interact with the above-mentioned proteins; we also included **AuL4** (a previously published complex sharing similar anti-tumor properties with **AuL7** but most active against HeLa cells) in these simulations, finding out that **AuL4** could interact with human topoisomerases and tubulin, as well, even though producing a different effect in the case of tubulin. To detail these interactions, we first performed two different tubulin assays and then we studied the inhibitory potential activity against human topoisomerases. The tubulin-polymerization assay demonstrated a clear reaction inhibition under **AuL7** exposure, whereas **AuL4** did not produce any inhibition but, rather, a curve similar to that of paclitaxel, known to increase tubulin polymerization. This early observation was elucidated using a specific immunostaining experiment, in which the inhibitory role in tubulin polymerization of **AuL7** was totally confirmed and the role of **AuL4** was definitely ascertained. Indeed, the **AuL4** treatment produced a stabilization of microtubules, in a similar way of the paclitaxel treatment, with the appearance of multinucleated cancer cells. Thus, even sharing the same metal and interacting with tubulin, the two complexes exhibited quite opposite effects on cytoskeleton dynamics, since **AuL7** is a microtubules disruptor and **AuL4** a microtubules stabilizer, at least under our experimental conditions. From in silico studies we argued that these effects could reside in the different ligands, bearing specific functional groups, used to coordinate Au, suggesting that the around chemical is responsible for the observed effects. These results are exciting because the combination of both complexes could induce a more powerful synergistic effect against the tumor growth. Next, we focused our attention towards the human topoisomerases, other important intracellular targets involved in uncontrolled cancer cells proliferation. Using an in vitro well-established test, we found out that both the **AuL7** and **AuL4** are selective and strong inhibitors of hTopo I and II, already at a concentration of 1 µM. The latter outcomes, together with the previous ones, makes these complexes effective multi-target drugs that affect unrelated intracellular proteins. Afterwards, we proved that **AuL7** induces the ROS-dependent intrinsic apoptotic pathway, with mitochondria damage and activation of caspases 3/7 and 9. Finally, cell cycle analysis demonstrated an increase in G2/M phase of MDA-MB-231 cells exposed to **AuL7**, which correlates well with the observed inhibition of both the hTopos and tubulin polymerization. Summing up, the most active complex, **AuL7**, represents a good lead candidate for further modifications turned to target other cell components and reinforce the already good anti-cancer properties.

## 4. Materials and Methods

### 4.1. Chemistry

All the reactions were carried out under nitrogen, using standard Schlenk or glovebox techniques. All the reagents were purchased from TCI Europe N.V. All commercially available chemicals in the experiments were of reagent grade and used as received. The solvents were purified according to the standard procedures. Deuterated solvents (Euriso–Top products) were degassed under a nitrogen flow and stored in the dark over activated 4Å molecular sieves. NMR spectra were recorded on a Bruker AM300 or a Bruker AVANCE 400 operating at 300 and 400 MHz for ^1^H and at 75 and 100 MHz for ^13^C, respectively. The ^1^H NMR and ^13^C NMR chemical shifts are referred to SiMe_4_ (δ = 0 ppm) by using the residual proton impurities of the deuterated solvents as internal standards. Chemical shifts, δ, were reported in parts per million (ppm) for both ^1^H and ^13^C NMR. Multiplicities are abbreviated as follows: singlet (s), doublet (d), triplet (t), quartet (q), multiplet (m), and broad (br). ^1^H COSY experiments allowed the assignment of all the proton resonances of the ^1^H NMR spectra, whereas Distortionless Enhancement by Polarization Transfer (DEPT) and Heteronuclear Multiple Bond Correlation (HMBC) experiments were useful for the attribution of ^13^C NMR signals. Mass spectra (ESI) were obtained by a Waters Quattro Micro triple quadrupole mass spectrometer equipped with an electrospray ion source. Elemental analyses for C, H, and N were recorded with a Thermo-Finnigan Flash EA 1112 and were performed according to standard microanalytical procedures. Chloride and iodide were determined indirectly by reaction of AgNO_3_ with halogen, precipitation of AgX (X = Cl, I), which was dissolved in Na_2_S_2_O_3_.

#### 4.1.1. Synthesis of N-substituted bis-imidazolium Salts (**L5**, **L6**)

*Synthesis of N-[4-hydroxymethyl-phenyl]-N’-[(2-hydroxy-2-phenyl)ethyl]-imidazolium bromide (L5)***:** Imidazole (2.10 g, 29 mmol) and styrene oxide (3.35 mL, 29 mmol, d = 1.054 g/L) in tetrahydrofuran (90 mL) were stirred for 12 h at 60 °C. The monoalkylated product obtained was not isolated. Then, 4-bromo-benzylalcohol (5.44 g, 29 mmol) was added. The solution was warmed to reflux for 24 h. The reaction mixture was cooled to room temperature and the solvent was removed in vacuo. At the crude product diethyl ether was added and filtered. The solution was extracted with water (twice 50 mL). The water was removed by evaporation in vacuo. The resulting residue was purified on a silica gel column chromatography in ethyl acetate/methanol (1/1) to give a yellow solid (38% yield).

^1^H NMR (300 MHz, CDCl_3_): 8.70 (s, 1H, NC*H*N), 7.58-6.95 (m, 11H, unsaturated protons), 5.24 (br,2H, PhC*H_2_*OH), 4.88 (dd, *J*(H,H)*_anti_* = 11.4,*J*(H,H)*_gauche_* = 5.7 Hz, 1H, C*H*OH), 4.14 (m, *J*(H,H) = 11.4, 5.7 Hz, 1H, NC*H*_2_), 4.07 (m, *J*(H,H) = 11.4, 5.7 Hz, 1H, NC*H*_2_). ^13^C NMR (75 MHz, CDCl_3_): 141.6, 137.5, 136.7, 135.0, 128.8, 128.4, 127.9, 126.8, 125.9, 120.1, 118.8, 72.6, 63.7, 54.7. Elemental analysis calcd (%) for C_18_H_19_N_2_O_2_Br: C, 57.61, H, 5.10, N, 7.47%; found: C, 57.29, H, 5.62, N, 7.59. ESI-MS (CH_3_CN, *m*/*z*): 188.1 Dalton attributable to [C_11_H_12_N_2_O]^+^.

*Synthesis of [N,N′-bis-[(2-hydroxy-2-phenyl)ethyl]-imidazolium iodide (L6):* Imidazole (1.0 g, 15 mmol) and styrene oxide (3.35 mL, 30 mmol, d = 1.054 g/L) in tetrahydrofuran (THF) (45 mL) were stirred for 24 h at 60 °C. The dialkylated product was purified by dissolution in acetone and obtained at low temperature (−20 °C) by precipitation. The intermediate was dissolved in CH_2_Cl_2_ (70 mL) and HI (stoichiometric amounts) was added to obtain white solid (43% yield).

^1^H NMR (300 MHz, DMSO-d_6_): 9.03 (s, 1H, NC*H*N), 7.67 (s, 1H), 7.63 (s, 1H), 7.37 (m, 10H, unsaturated protons), 5.93 (br, 2H, O*H*), 4.96 (m, *J*(H,H) = 8.7, 2.6 Hz,2H, 2·C*H*OH), 4.44 (dd, *J*(H,H) = 5.7, 11,4 Hz, 2H, 2·NC*H*_2_) 4.28 (dd, *J*(H,H) = 5.7, 11,4 Hz,2H, 2·NC*H*_2_). ^13^C NMR (75 MHz, DMSO-d_6_): 141.2, 137.5, 127.9, 127.6, 127.2, 125.9, 119.8, 71.9, 53.4. Elemental analysis calcd (%) for C_19_H_21_N_2_O_2_I: C, 52.31, H, 4.85, N, 6.42%; found: C, 52.68, H, 4.82, N, 6.38%. ESI-MS (CH_3_CN, *m*/*z*): 309.1 Dalton attributable to [C_19_H_21_N_2_O_2_]^+^.

#### 4.1.2. Synthesis of Complexes **AgL5**, **AuL5**, **AgL6**, and **AuL6**

Silver and gold complexes were prepared by using a slightly modified literature procedure [10,11].

*Synthesis of N-[4-(hydroxymethyl)phenyl]-N’-[(2-hydroxy-2-phenyl)ethyl]-imidazole-2-ylidene]-silver(I)bromide] (AgL5):* Ligand **L5** (0.250 g, 0.66 mmol) was added to a suspension of silver oxide (0.308 g, 1.33 mmol) in CH_2_Cl_2_ (15 mL) containing molecular sieves (4 Å). The reaction was carried out with the exclusion of light. The mixture was heated at reflux for 1.5 h. Then, the mixture was filtered on celite and solvent was removed under high vacuum, the residue washed with hexane and dried in vacuo to give compound **AgL5** as a light brown powder (49.5% yield).

^1^H NMR (400 MHz, CD_2_Cl_2_): 7.54–6.89 (m, 11H, unsaturated carbons), 5.29 (br, 2H, PhC*H_2_*OH), 4.92 (dd, 1H, C*H*OH), 4.20 (m, 1H, NC*H*_2_), 4.10 (m, 1H, NC*H*_2_). ^13^C NMR (100 MHz, CDCl_3_): 137.9, 137.3, 137.0, 129.3, 127.0, 126.0, 120.0, 118.7, 73.7, 64.8, 63.7, 54.9. Elemental analysis calcd (%) for C_18_H_18_N_2_O_2_AgBr: C, 44.84, H, 3.76, N, 5.81%; found: C, 44.28, H, 4.07, N, 5.89. ESI-MS (CH_3_CN, *m*/*z*): 485.8 - 483.0 Dalton are attributable to [C_18_H_19_AgBrN_2_O_2_]^+^.

*Synthesis of N-[4-(hydroxymethyl)phenyl]-N’-(2-hydroxy-2-phenyl)ethyl]-imidazole-2-ylidene]-gold(I)bromide](AuL5):N*-[4-(hydroxymethyl)phenyl]-*N*’-(2-hydroxy-2-phenyl)ethyl]-imidazole-2-ylidene]-gold(I)- bromide] (**AuL5**)was prepared by trans-metalation from **AgL5**. The silver complex (0.150 g, 0.031 mmol) and (CH_3_)_2_SAuCl (0.091 g, 0.031 mmol) were suspended in 23 mL of dichloromethane and allowed to stir at room temperature for 12 h in the absence of light. The resulting suspension was then passed through a Celite pad. The solvent was concentrated and then treated with an excess of hexane to precipitate as a violet powder (15% yield).

^1^H NMR (400 MHz, DMSO-d_6_): 8.03 (s, 1H, C*H*CH), 7.72 (s,1H, CHC*H*), 7.44-7.04 (m, 9H, aromatic protons), 5.85–5.35 (br, 2H, PhC*H_2_*OH), 4.91 (br, 1H, NC*H*_2_), 4.23–3.97 (br, 2H, NC*H*_2_). ^13^CNMR (100 MHz, DMSO-d_6_): 161.2 (N*C*N), 141.8, 141.7, 140.9, 140.4, 140.2, 139.6, 139.0, 137.1, 136.9 137.96, 137.36, 137.01, 128.8, 128.5, 128.4, 128.2, 127.6, 127.0, 128.9, 122.0, 121.7, 120.7, 120.4, 71.1, 64.0, 62.3, 54.7. Elemental analysis calcd (%) for C_18_H_18_N_2_O_2_AuBr: C, 37.78, H, 4.90, N, 5.32%; found: C, 37.56, H, 4.68, N, 5.41.ESI-MS (CH_3_CN, *m*/*z*): 571.3 and 573.0 Dalton attributable to [C_18_H_19_AuBrN_2_O_2_]^+^.

*Synthesis of [bis-N,N′-(2-hydroxy-2-phenyl)ethyl]-imidazole-2-ylidene]-silver(I)]^+^[diiodide-silver(I)]^−^ (AgL6):***AgL6** was obtained by reaction of **L6** (0.20 g, 0.65 mmol) and Ag_2_O (0.09 g, 0.37 mmol) in 41 mL acetonitrile at reflux for 12 h leading to the silver (I) complex. After reaction, the mixture was filtered on celite and solvent was removed, the residue washed with hexane and dried under high vacuum to gave compound AgL6 as a yellow powder (50%, yield).

^1^H NMR(300 MHz, DMSO-d_6_): 7.47–7.32 (m, 5H, aromatic protons), 7. 09 (s, 1H, C*H*CH), 6.80 (*s, 1H,* CHC*H*), 5.70 (br, 1H, O*H*), 4.80 (m, 1H, C*H*OH), 4.10 (dd, 1H, NC*H*_2_), 4.01 (dd, 1H, NC*H*_2_). ^13^CNMR (75 MHz, DMSO-d_6_): 143.1, 138.1, 128.5, 128.1, 127.9, 127.7, 126.5, 126.2, 120.4, 72.56, 54.0. Elemental analysis calcd (%) for C_19_H_20_N_2_O_2_AgI: C, 42.02, H, 3.71, N, 5.16%, I, 23.30%; found: C, 42.05, H, 3.79, N, 5.07%, I, 23.45%. ESI-MS (CH_3_CN, *m*/*z*): 482.8 and 485.3 Dalton attributable to [C_22_H_23_AgN_4_O_2_]^+^.

*Synthesis of [bis-N,N′-(2-hydroxy-2-phenyl)ethyl]-imidazole-2-ylidene]-gold(I)]^+^[dichloride-gold(I)]^−^(AuL6):* KHMDS [potassium-bis-(trimethylsilyl)-amide] (0.097 g, 0.48 mmol) was added to a solution of **L6** (0.15 g, 0.48 mmol) in DMF (7 mL). The resulting orange solution was stirred for 15 min and a solution of (CH_3_)_2_SAuCl (0.12 g, 0.38 mmol) in DMF (2 mL) was added dropwise. The mixture was stirred at room temperature for 3 h. The solvent was removed under vacuum. The resulting dark yellow solid was washed with CH_2_Cl_2_ (3 × 10 mL) and dried under vacuum to gave compound **AuL6** (55.9% yield).

^1^H NMR(300 MHz, DMSO-d_6_): 7.63–7.32 (m, 7H, aromatic protons), 5.84 (br, 1H, O*H*), 4.90 (m, 1H, C*H*OH), 4.29 (dd, 1H, NC*H*_2_), 4.16 (dd, 1H, NC*H*_2_). ^13^C NMR (75 MHz, DMSO-d_6_): 162.2, 142.1, 128.2, 127.53, 126.0, 71.5, 54.9. Elemental analysis calcd (%) for C_19_H_20_N_2_O_2_AuCl: C, 42.20, H, 3.73, N, 5.18%, Cl, 6.54%; found: C, 42.01, H, 3.62, N, 5.28%, Cl, 6.43%. ESI-MS (CH_3_CN, *m*/*z*): 669.0 Dalton attributable to [C_32_H_32_AuN_4_]^+^.

### 4.2. Docking Simulations

The crystal structures of the polymeric complex formed between tubulin α-1B and tubulin β-2B chains, Stathmin 4 and Tubulin Tyrosine Ligase (PDB code 5J2T) [64], of the human topoisomerase I in covalent and noncovalent complexes with DNA (PDB code 1A35) [65], and of *Saccharomyces cerevisiae* topoisomerase II in complex with a short DNA fragment and the ATP-analog AMPPNP (PDB Code 4GFH) [66] have been used as templates to build the complete three-dimensional models of human tubulin, topoisomerase I (hTopo I) and topoisomerase II (hTopoII), as previously described [28,67]. All the ligand structures tested in silico have been built and energy minimized using the program MarvinSketch [ChemAxon ltd, Budapest, Hu]. To evaluate the possible binding modes and the binding energies of different derivatives to the three proteins, we used the Autodock v.4.2.2. [68] program suite. We performed a “blind-docking” simulation: the docking of the small molecule to the targets was done without a priori knowledge of the location of the binding site by the system. All the simulations were done using the standard default values. The protein and the ligands were prepared using the ADT graphical interface [69]. Polar hydrogens were added to each protein, Kollman charged assigned and eventually solvatation parameters added. Each protein was considered to be a rigid object while all the ligands as fully flexible. A searching grid was extended all over the protein and affinity maps calculated. The search was carried out with a Lamarckian Genetic Algorithm: a population of 100 individuals with a mutation rate of 0.02 were evolved for 100 generations. Evaluation of the results was performed by listing the different ligand poses accordingly to their predicted binding energy. A cluster analysis based on root mean squares deviation (RMSD) values from the starting geometry was performed. The lowest energetic conformation of the most populated cluster was consider as the best candidate. When clusters are almost equipopulated and their energy distribution is spread, their corresponding molecules were considered to be bad ligands [70,71].

The generated docking poses were ranked in order of increasing binding energy values and clustered on the basis of a RMSD cut-off value of 2.0 Å. From the structural analysis of the lowest energy solutions of each cluster, we could spot the protein binding site. Figures were drawn using the program Chimera [72].

### 4.3. Biology

#### 4.3.1. Cell Culture

The six cell lines used in this work (MCF-7, MDA-MB-231, HeLa, ISHIKAWA, MCF-10A and Hek-293) were purchased from American Type Culture Collection (ATCC, Manassas, VA, USA). MCF-7 human breast cancer cells, estrogen receptor (ER)-positive, were maintained in Dulbecco’s modified Eagle’s medium/nutrient mixture Ham F-12 (DMEM/F12), supplemented with 10% fetal bovine serum (FBS) and 100 U mL^−1^ penicillin/streptomycin. MCF-10A human mammary epithelial cells, were cultured in DMEM/F12 medium, supplemented with 5% horse serum (HS, Thermo Fisher Scientific, Milan, Italy), 100 U mL^−1^ penicillin/streptomycin, 0.5 mg mL^−1^ hydrocortisone, 20 ng mL^−1^ human epidermal growth factor (hEGF), 10 mg mL^−1^ insulin, and 0.1 mg mL^−1^ cholera enterotoxin (Sigma–Aldrich, Milan, Italy). MDA-MB-231 human breast cancer cells, known as triple-negative cells (i.e., not overexpressing human epidermal growth factor receptor 2 (HER2), estrogen and progesterone receptors), were cultured in DMEM/F12 supplemented with 5% FBS, 1% l-glutamine and 100 U mL^−1^ penicillin/streptomycin. HeLa human epithelial cervix carcinoma cells, estrogen receptor (ER)-negative, and ISHIKAWA human endometrial adenocarcinoma cells, estrogen receptor (ER)-positive, were maintained in minimum essential Eagle’s Medium (MEM), supplemented with 10% FBS, 1% l-glutamine, 100 U mL^−1^ penicillin/streptomycin and 1% Non-Essential Amino Acids (NEAA). Hek-293 human embryonic kidney epithelial cells were cultured in DMEM high glucose (4.5 g L^−1^), supplemented with 10% FBS, 1% l-glutamine and 100 U mL^–1^ penicillin/streptomycin. Cells were maintained at 37 °C in a humidified atmosphere of 95% air and 5% CO_2_ and periodically screened for contamination [37].

#### 4.3.2. Cell Viability

Cell viability has been determined using the 3-(4,5-dimethylthiazol-2-y1)-2,5-diphenyltetrazolium bromide (MTT, Sigma–Aldrich, Milan, Italy) assay [73,74]. Cells have been seeded on 48-well plates and grown in complete medium. Before being treated, cells have been starved in serum free medium for 24 h for allowing cell cycle synchronization. Then cells were treated in phenol-red-free medium supplemented with 1% of serum with increasing concentrations of each compound for 72 h and after fresh MTT, re-suspended in phosphate-buffered saline (PBS), was added to each well (final concentration (0.5 mg/mL). After 2 h incubation at 37 °C, cells have been lysed with DMSO, and then optical density was measured at 570 nm using a microplate reader. For each sample, the mean absorbance was expressed as a percentage over the control and plotted versus drug concentrations to determine the IC_50_ values (i.e., drug concentrations able to decrease cell viability by 50% with respect to control) for each cell line, using GraphPad Prism 6 software (GraphPad Inc., San Diego, CA, USA). Data are representative of three independent experiments; standard deviations (SD) have been shown.

#### 4.3.3. Detection of Intracellular H_2_O_2_ (ROS)

Cells were grown on glass coverslips until the treatment with compound or menadione (used as positive control) (Sigma–Aldrich, Milan, Italy). Then, cells were incubated with 10 μM 2′-7′-Dichlorofluorescein diacetate (Sigma–Aldrich, Milan, Italy) for 40 min at 37 °C in a 5% CO_2_ incubator, as previously described [28]. Then, DAPI (0.2 μg mL^−1^) counterstain was performed for 10 min at room temperature, in the dark. Cells were then washed three times with cold PBS, adding one drop of mounting solution, then they were observed and imaged under a fluorescence microscope (Leica DM6000; 20× magnification) with excitation/emission wavelength maxima of 490 nm/515 nm (DCF) or 350 nm/460 nm (DAPI). Images are representative of three independent experiments.

#### 4.3.4. TUNEL Assay

Apoptosis was detected by the TUNEL assay, according to the guidelines of the manufacturer (CF^TM^488A TUNEL Assay Apoptosis Detection Kit, Biotium, Hayward, CA, USA), with some modifications [37]. In brief, cells were grown on glass coverslips until they were treated with compounds to test. After the treatment, they were washed three times with PBS, then methanol fixed at −20 °C for 15 min. Fixed cells were incubated with enzyme, as previously described [75]. At the end, DAPI (0.2 mg mL^−1^) counterstain was performed for 10 min at room temperature, protected from light. Fixed cells were then washed three times with cold PBS, added with one drop of mounting solution, observed and imaged under a fluorescence microscope (Leica DM6000; 20× magnification) with excitation/emission wavelength maxima of 490 nm/515 nm (CF^TM^488A) or 350 nm/460 nm (DAPI) [76]. Images are representative of three independent experiments.

#### 4.3.5. Caspase Assay

Caspases-3/7, -8, and -9 activities were measured with the caspase-Glo Assay, according to the guidelines of manufacturer (Caspase-Glo^®^ 3/7, 8 and 9 Assay Systems, Promega Corporation, Madison, WI, USA). Cells were grown in white-walled 96-well plates and, after treatment, 100 µL of different caspase was added to each well as previously described in Iacopetta et al. [11].

#### 4.3.6. Cytoskeleton and Mitochondrial Staining and Immunofluorescence

Cells were grown on glass coverslips in full media, then serum-deprived for 24 h and exposed to compounds for 24 h (concentration equal to its IC_50_ value). Then, the cells after PBS-washed, were fixed with cold methanol (15 min/−20 °C) and incubated with primary antibody, diluted in blocking solution (4 °C/overnight), as previously described [76]. The mouse anti-β-Actin (69879) and the rabbit anti-β tubulin (9104) were purchased from Santa Cruz Biotechnology and used at 1:100 dilution. Coverslips were then washed 3 times with PBS, then fixed cells were incubated with the secondary antibody Alexa Fluor^®^ 568 conjugate goat-anti-mouse and Alexa Fluor^®^ 488 conjugate goat-anti-rabbit (1:500, Thermo Fisher Scientific, Waltham, MA, USA). Nuclei were stained using DAPI (Sigma–Aldrich, Mila, Italy) for 10 min at a concentration of 0.2 μg/mL then washed 3 times with PBS. Fluorescence was detected using a fluorescence microscope (Leica DM 6000). LAS-X software was used to acquire and process all images [77].

The same protocol was performed for mitochondrial staining. However, in the initial phase of this assay, the cells were pre-incubated with pre-warmed (37 °C) staining solution containing MitoTracker^®^ Deep Red FM probe (MitoTracker^®^ Mitochondrion-Selective Probes, Invitrogen European Headquarters, Paisley PA4 9RF, UK) for 20 min (Fluorescence excitation = 644, Fluorescence emission = 665). The primary antibody used in this case was the mouse anti-cytochrome c (556 433), purchased from BD Biosciences. The secondary antibodies used was Alexa Fluor^®^ 488 conjugate goat-anti-mouse (1:500 dilution), purchased from Thermo Fisher Scientific, Waltham, MA, USA.

#### 4.3.7. In Vitro Tubulin-Polymerization Assay

Tubulin-polymerization inihibition was measured using in vitro Tubulin-Polymerization Assay Kit purchased fromEMD Millipore Corporation. Polymerization reactions occur in 70 µL final volumes, of which 60 µL is the 60 µM tubulin in 1 × PB-GTP and 10 µL is the test substance dissolved in 1 × PB-GTP. Paclitaxel, vinblastine (used as control) and **AuL7**, were dissolved in DMSO and used at final concentration of 10 µM. These reactives were combined in 96-well plate on ice. After the plate was transferred into the spectrophotometer pre-warmed at 37 °C and the turbidity variation was measured every 30 s at 350 nm for 90 min. The plate was shaked for 10 s before each measurement. Turbidity (absorbance) readings were used to calculate the extent of polymerization [% inhibition = (1−A_350_ sample/A_350_ control) × 100].

#### 4.3.8. Human Topoisomerase I Relaxation Assay

hTopo I relaxation assays have been performed in a final volume of 20 mL, as reported by Iacopetta et al. [37,78], incubating 0.25 mg of supercoiled plasmid pHOT1 in Tris-EDTA (TE) buffer (TopoGEN, Port Orange, FL, USA) with tested compounds and recombinant hTopo I (2 units) (TopoGEN, Port Orange, FL, USA) for 1h at 37 °C. The obtained solution has been loaded in a 1% agarose gel containing 1× Tris-acetate-EDTA (TAE) buffer without ethidium bromide (EB). Agarose gel has been stained using 1× TAE buffer with EB (0.5 mg/mL) for 30 min and after washed with distilled water for 15 min. At the end, it has been visualized by using a ultraviolet (UV) transilluminator. The experiment was performed in triplicate.

#### 4.3.9. Human Topoisomerase II Decatenation Assay

hTopo II decatenation assays were performed in a final volume of 20 mL, as reported by Iacopetta et al. [37,78], using 0.2 mg of kinetoplast DNA (kDNA) (topoGEN, Port Orange, FL, USA), tested compounds and 3 units of hTopo II (topoGEN, Port Orange, FL, USA). The reaction products were loaded in an agarose gel with EB. At the end, the gel was washed with distilled water and analyzed using a UV transilluminator. The experiment was repeated three times.

#### 4.3.10. Cell Cycle

MDA-MB-231 cells were seeded in a 6-well plate (EuroClone, Milan, Italy) at a density of 1.8 × 10^5^ cells/well. The next day the cells were treated with compound **AuL7** at a final concentration equal to its IC_50_ for 72 h. To compare the results induced by **AuL7**, we also treated MDA-MB-231 cells with the same amount of DMSO but without **AuL7** compound. At the end of the incubation, we fixed all the treated cells and performed Muse Cell Cycle Assay as suggested by the manufacturer (EMD Millipore Corporation, Billerica, MA, USA).

#### 4.3.11. Statistical Analysis

Data were analyzed for statistical significance (*p* < 0.001) using one-way ANOVA followed by Dunnett’s test performed by GraphPad Prism 6. SDs are shown.

## Figures and Tables

**Table 1:** IC_50_ values of **L5**, **L6**, and **L7** metal complexes and Cisplatin, expressed in μM. The means ± standard deviations are shown.

IC_50_ (µM)						
Compounds	MCF-7	MDA-MB-231	HeLa	ISHIKAWA	MCF-10A	Hek-293
**L5**	>200	>200	>200	>200	>200	>200
**AgL5**	>200	>200	>200	>200	>200	>200
**AuL5**	18.2 ± 0.5	31.7 ± 0.8	44.5 ± 0.9	54.9 ± 1.0	>200	>200
**L6**	>200	>200	>200	>200	>200	>200
**AgL6**	29.7 ± 0.3	57.9 ± 0.5	47.7 ± 0.4	26.7 ± 0.5	143.4 ± 0.8	>200
**AuL6**	9.38 ± 0.2	53.7 ± 0.7	98.6 ± 1.2	12.6 ± 0.3	158.8 ± 1.0	>200
**L7**	>200	>200	>200	>200	>200	>200
**AgL7**	10.5 ± 0.8	3.22 ± 1.2	9.76 ± 0.6	6.55 ± 0.9	>200	>200
**AuL7**	5.18 ± 0.4	2.1 ± 0.7	31.9 ± 0.5	29.9 ± 0.9	>200	65.0 ± 0.7
**AuL7 +** **Quercetin** (4 h)	-	-	-	-	-	96.9 ± 1.1
***Cis-*platin**	35.8 ± 1.3	28.7 ± 1.0	15.7 ± 1.1	15.1 ± 0.8	81.3 ± 1.2	16.8 ± 0.4

**Table 2:** Binding Energies and Binding constants for each compound tested in silico towards human topoisomerases I and II and tubulin.

Compounds	Tubulin		Topoisomerase I		Topoisomerase II	
	E_b_ (kcal/mol)	K_i_ (μM)	E_b_ (kcal/mol)	K_i_ (μM)	E_b_ (kcal/mol)	K_i_ (μM)
**AuL4**	−7.4	3.78	−5.83	53.0	−6.39	20.86
**AgL5**	−7.66	2.43	−7.44	3.51	−7.72	2.21
**AuL5**	−8.45	0.6629	−7.55	2.92	−7.49	3.03
**AgL6**	−7.73	2.17	−7.73	2.17	−6.44	18.91
**AuL6**	−6.76	8.26	−8.9	0.3219	−7.84	4.16
**AgL7**	−13.27	1.3 × 10^−6^	−9.18	0.1853	−10.06	0.0423
**AuL7**	−11.3	0.0052	−8.42	0.6694	−6.84	9.64
